# Improved simultaneous co-fermentation of glucose and xylose by *Saccharomyces cerevisiae* for efficient lignocellulosic biorefinery

**DOI:** 10.1186/s13068-019-1641-2

**Published:** 2020-01-22

**Authors:** Phuong Hoang Nguyen Tran, Ja Kyong Ko, Gyeongtaek Gong, Youngsoon Um, Sun-Mi Lee

**Affiliations:** 1 Clean Energy Research Center, Korea Institute of Science and Technology (KIST), Seoul, 02792 Republic of Korea; 2 Division of Energy and Environment Technology, University of Science and Technology (UST), Daejeon, 34113 Republic of Korea; 3 Green School, Korea University, Seoul, 02841 Republic of Korea

**Keywords:** Lignocellulosic biorefinery, Efficient co-fermentation, *Saccharomyces cerevisiae*, Xylose isomerase, Bioethanol

## Abstract

**Background:**

Lignocellulosic biorefinery offers economical and sustainable production of fuels and chemicals. *Saccharomyces cerevisiae*, a promising industrial host for biorefinery, has been intensively developed to expand its product profile. However, the sequential and slow conversion of xylose into target products remains one of the main challenges for realizing efficient industrial lignocellulosic biorefinery.

**Results:**

In this study, we developed a powerful mixed-sugar co-fermenting strain of *S. cerevisiae*, XUSEA, with improved xylose conversion capacity during simultaneous glucose/xylose co-fermentation. To reinforce xylose catabolism, the overexpression target in the pentose phosphate pathway was selected using a DNA assembler method and overexpressed increasing xylose consumption and ethanol production by twofold. The performance of the newly engineered strain with improved xylose catabolism was further boosted by elevating fermentation temperature and thus significantly reduced the co-fermentation time by half. Through combined efforts of reinforcing the pathway of xylose catabolism and elevating the fermentation temperature, XUSEA achieved simultaneous co-fermentation of lignocellulosic hydrolysates, composed of 39.6 g L^−1^ glucose and 23.1 g L^−1^ xylose, within 24 h producing 30.1 g L^−1^ ethanol with a yield of 0.48 g g^−1^.

**Conclusions:**

Owing to its superior co-fermentation performance and ability for further engineering, XUSEA has potential as a platform in a lignocellulosic biorefinery toward realizing a more economical and sustainable process for large-scale bioethanol production.

## Background

Lignocellulosic biomass is considered as an economical and sustainable feedstock for the production of fuels and chemicals via microbial fermentation. *Saccharomyces cerevisiae* is regarded as one of the most promising industrial hosts for biorefinery, with well-developed genetic tools and proven industrial feasibility, and it has been intensively engineered to realize microbial production of various fuels and chemicals in biorefinery concept [1]. One of the main challenges to achieving an economically feasible and competitive lignocellulosic biorefinery with expanded product profile is realizing the complete bioconversion of all available sugars in the lignocellulosic biomass. Therefore, developing an *S. cerevisiae* strain with high capacity for the simultaneous co-fermentation of glucose and xylose, the two most abundant sugars derived from lignocellulosic hydrolysates [2], has attracted substantial attention in recent years.

Through extensive efforts in metabolic and evolutionary engineering, recombinant *S. cerevisiae* is now able to convert xylose into ethanol as the sole carbon source [3]. However, even a strain with efficient xylose catabolism cannot necessarily perform the simultaneous co-fermentation of glucose and xylose owing to a limited xylose conversion rate in the presence of glucose, which is a major remaining challenge for achieving the efficient bioconversion of lignocellulosic biomass into biofuels with engineered strains of *S. cerevisiae* [1]. This limitation is mainly due to the deterioration in xylose utilization efficiency during co-fermentation. Therefore, to overcome this problem, transporter engineering has been applied to boost the xylose import into cells by introducing heterologous pentose transporters or overexpressing homologous pentose-switchable hexose transporters [4–7]. Nevertheless, the co-fermentation performance of transporter-engineered *S. cerevisiae* strains in the sequential utilization of glucose and xylose remains suboptimal despite significantly increasing xylose uptake [8, 9].

Recently, the successful simultaneous fermentation of glucose and xylose was reported using engineered xylose-utilizing strains with an isomerase-based pathway even without transporter engineering. With isomerase-based pathway, not only high-yield bioethanol production was achieved owing to the cofactor-neutral nature, but also simultaneous utilization of glucose and xylose was realized [10–12]. However, the glucose utilization rate with these strains is still much faster than that of xylose due to the limited metabolic flux through isomerase-based xylose catabolic pathway, so that further improvement in the xylose utilization efficiency in isomerase-based xylose-utilizing *S. cerevisiae* is required to truly realize the efficient co-fermentation of glucose and xylose for an economically feasible lignocellulosic biorefinery.

In engineered *S. cerevisiae* harboring the isomerase-based pathway, bioconversion of xylose is initiated with the isomerization of xylose to xylulose, which is catalyzed by xylose isomerase through three successive reactions of ring-opening, isomerization, and ring-forming [13]. These isomerization steps are similar to those involved in glucose isomerization, which is an endothermic reaction based on its reaction enthalpy [14]. According to Le Chatelier’s principle, increasing the temperature of the reaction system will shift the equilibrium in the direction of the endothermic reaction. Therefore, we hypothesized that increasing the fermentation temperature would enhance the isomerization of xylose into xylulose and thus improve the xylose utilization efficiency in an engineered *S. cerevisiae* with an isomerase-based pathway. This beneficial effect of increased reaction temperature on the enzymatic isomerization of xylose was previously proven in vitro over a wide temperature range (25–45 °C) [15]. However, the impact of elevating the temperature for fermentation on the productivity of a xylose-utilizing strain of *S. cerevisiae* harboring an isomerase pathway has not yet been evaluated.

Therefore, in the present study, we sought to improve glucose/xylose co-fermentation efficiency of *S. cerevisiae* through the synergistic effects of enhanced xylose catabolism and elevating fermentation temperature. Previously, we developed an efficient glucose and xylose co-fermenting strain, XUSE, capable of high-yield ethanol production and simultaneous glucose/xylose fermentation with negligible inhibition of glucose [12]. To boost up the xylose catabolism in XUSE, we reinforced xylose catabolism by overexpressing a selected gene target in the pentose phosphate pathway (PP pathway), of which all the involved genes are routinely overexpressed to develop xylose-utilizing strains [16, 17], by harnessing the power of a DNA assembler method [18] and growth-based selection strategy. We further improved xylose conversion rates by elevating fermentation temperature based on the endothermic nature of xylose isomerization in the initial xylose catabolic pathway in XUSEA. The co-fermentation efficiency of XUSEA was then evaluated in terms of ethanol yield and xylose consumption rates during lignocellulosic bioethanol production. Consequently, this study provides a promising platform host for lignocellulosic biorefinery that can achieve economically feasible and sustainable production of fuels and chemicals with high titer, yield and productivity.

## Results

### Development of an efficient glucose and xylose co-fermenting *S. cerevisiae* strain

**Fig. 1:**
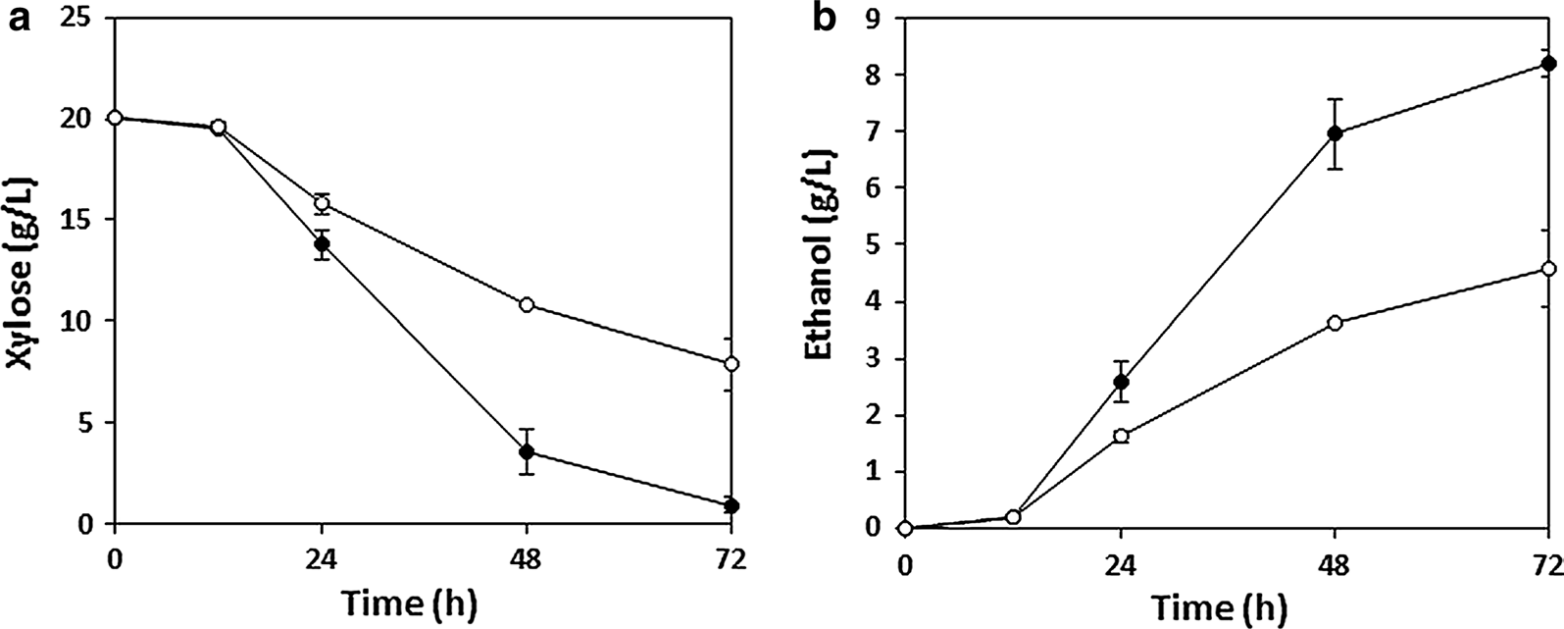
Fermentation performance of xylose (20 g L^−1^) between two strains: XUSE expressing pRPE1 vector (black) and XUSE expressing empty vector (white). **a** Xylose consumption rate, and **b** ethanol production rate. Error bars represent the standard deviation of biological triplicates

We sought to enhance xylose catabolism in our previously engineered *S. cerevisiae* strain XUSE by overexpressing the genes involved in the PP pathway. To this end, we first tried to find the most effective combination of genes involved in the PP pathway that would improve xylose catabolism in XUSE while minimizing the burden on the cells caused by unnecessary overexpression. Screening of cells randomly expressing genes in the PP pathway at different combinations allowed for selection of strains showing rapid growth on xylose (Additional file 1: Figure S1); the best-performing strains were those expressing *RPE1*. Specifically, with overexpression of *RPE1*, XUSE exhibited almost double the amount of xylose utilization and ethanol production during 72 h of xylose fermentation (Fig. 1). To further improve the xylose conversion efficiency in XUSE, we decided to integrate one copy of the xylA*3 and *RPE1* genes each into the *ASC1* locus using the marker-free CRISPR-Cas9 genome editing system, generating the new strain XUSEA. In our previous study, whole-genome sequencing of XUSE identified a mutation on *ASC1*^Q237*^, which seemed to cause the loss of function of ASC1, and this could offer an integration site for further strain engineering without causing phenotypic changes [12].

Boosting up the metabolic flux of XUSE through the xylose catabolic pathway by adding an additional copy of xylose isomerase and increasing metabolic flux through the PP pathway via *RPE1* overexpression resulted in significantly improved xylose utilization in our newly developed strain XUSEA compared to that of XUSE. During fermentation with a low cell density, XUSEA completely consumed 19.6 g L^−1^ xylose within 72 h to produce 9 g L^−1^ ethanol with a yield of 0.46 g g^−1^, while XUSE converted 18.7 g L^−1^ of xylose into 8.3 g L^−1^ ethanol with a yield of 0.44 g g^−1^ (Additional file 2: Figure S2). The overall xylose conversion rate and ethanol productivity of XUSEA were 0.39 g xylose g DCW^−1^ h^−1^ and 0.17 g ethanol g DCW^−1^ h^−1^, respectively, representing an increase of 26% and 21%, respectively, from those obtained with XUSE (0.31 g xylose g^−1^ h^−1^ and 0.14 g ethanol g^−1^ h^−1^).

**Fig. 2:**
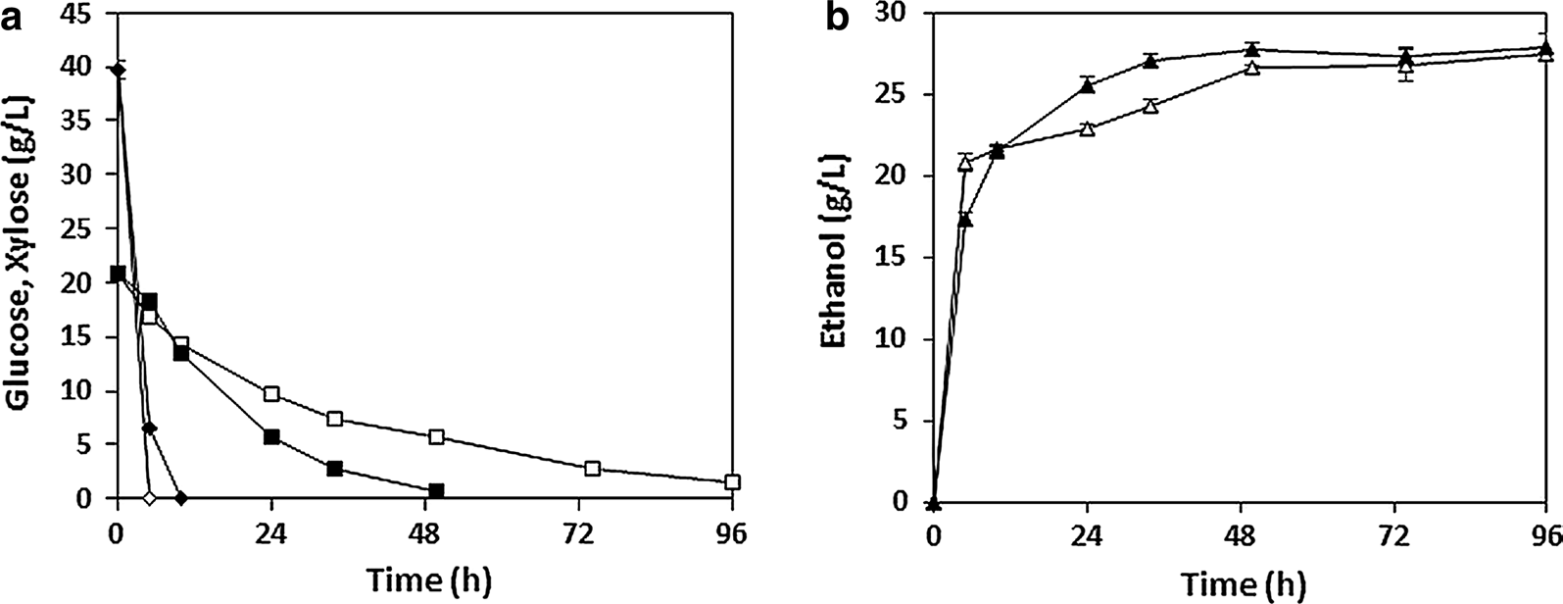
Micro-aerobic co-fermentation of glucose (40 g L^−1^) and xylose (20 g L^−1^) with the XUSEA (black) and XUSE (white) strains at a high cell density (initial OD_600_ = 20). **a** Glucose (open rhombus) and xylose (open square) consumption, **b** ethanol (open triangle) production. Error bars represent the standard deviation of biological triplicates

The improved xylose fermentation performance of XUSEA was more clearly demonstrated during high-cell-density co-fermentation of glucose and xylose (Fig. 2). During co-fermentation of 40 g L^−1^ glucose and 20 g L^−1^ xylose, both XUSE and XUSEA showed the simultaneous utilization of glucose and xylose. However, owing to its improved xylose utilization capacity, the total fermentation time required for XUSEA was remarkably reduced compared to that required for XUSE. XUSE required 96 h to convert all the glucose and xylose into ethanol, whereas XUSEA completely utilized all sugars within only 50 h, demonstrating the same level of activity in about half the time. During co-fermentation, XUSEA produced 27.7 g L^−1^ of ethanol with an ethanol yield of 0.46 g g^−1^, verifying its superior co-fermentation performance over that of the XUSE strain.

### Fermentation performance was maintained even with high-level mixed sugars

**Fig. 3:**
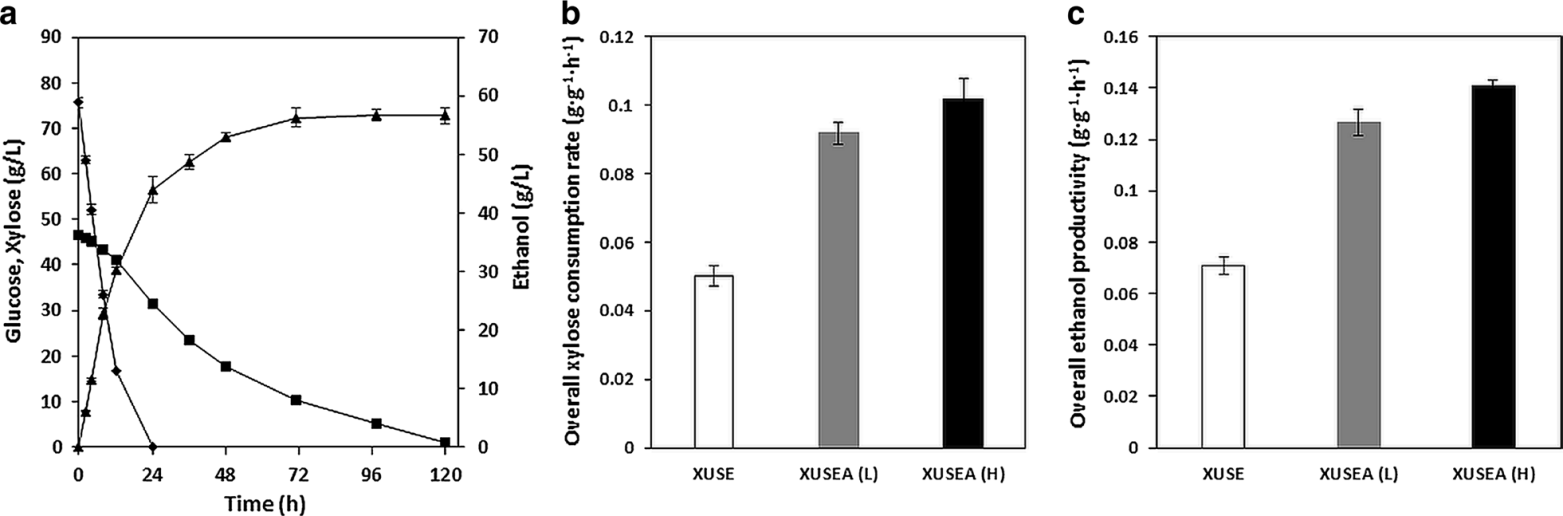
Micro-aerobic co-fermentation performance of XUSEA with a high level of mixed sugars (76 g L^−1^ glucose and 46 g L^−1^ xylose) at high cell density (initial OD_600_ of 20). **a** sugar consumption and ethanol production: (filled rhombus) glucose, (filled square) xylose, (filled triangle) ethanol. **b** overall xylose consumption rate and **c** overall ethanol productivity of XUSE, XUSEA at a low (L) and a high (H) level of mixed sugars. Xylose consumption and ethanol production rates of XUSE are obtained with a low level of mixed sugars (40 g L^−1^ glucose and 20 g L^−1^ xylose). Error bars represent the standard deviation of biological triplicates

To evaluate the co-fermentation performance of XUSEA in an industrial setting, we conducted high-level mixed-sugar fermentation with 76 g L^−1^ of glucose and 46 g L^−1^ of xylose, which are considered the target ranges of sugar concentrations for an industrial-scale ethanol production process to achieve economic feasibility [11, 19]. XUSEA presented a maximal ethanol titer of 56.7 g L^−1^, reaching a yield of 0.5 g g^−1^ at 72 h (Fig. 3). The overall xylose and total sugar consumption rates, and ethanol productivity were 0.1 g xylose g cell^−1^ h^−1^, 0.29 g total sugars g cell^−1^ h^−1^, and 0.14 g ethanol g cell^−1^ h^−1^, respectively. With increased sugar concentrations, both the xylose and total sugar consumption rates, and ethanol productivity slightly improved compared to those obtained during fermentation with 40 g L^−1^ of glucose and 20 g L^−1^ of xylose (0.09 g xylose g^−1^ h^−1^, 0.27 g total sugars g^−1^ h^−1^ and 0.13 g ethanol g^−1^ h^−1^, respectively) (Fig. 3). This indicates that the co-fermentation performance was not inhibited by a high concentration of sugars and highlights the potential of XUSEA as a promising platform host for the commercial production of lignocellulosic bioethanol. Even with a high glucose concentration, XUSEA simultaneously consumed both glucose and xylose without glucose repression on xylose utilization (Fig. 3). To our knowledge, XUSEA shows the highest ethanol titer and yield, 56.7 g L^−1^ and 0.48 g g^−1^, respectively, among those of previously reported strains with a similar high-level sugar mixture (Table 1).

**Table 1:** Comparison of the co-fermentation performance of XUSEA with those of previously reported recombinant xylose-utilizing *S. cerevisiae* strains in a high-level mixed synthetic sugar medium

Strain	Description	Culture medium	Glucose (g L^−1^)	Xylose (g L^−1^)	Overall xylose consumption rate (g g^−1^ h^−1^)	Overall total sugar consumption rate (g g^−1^ h^−1^)	Max. ethanol concentration (g L^−1^)	Overall ethanol productivity (g g^−1^ h^−1^)	Ethanol yield (g g^−1^)	References
XUSEA^a^	BY4741, xylA*3, TAL1, XKS1, RPE1, *Δ*gre3, *Δ*pho13, *Δ*asc1, evolved	Defined CSM medium	39.6	22.8	0.22	0.61	29.1	0.28	0.47	This study
XUSEA	BY4741, xylA*3, TAL1, XKS1, RPE1, *Δ*gre3, *Δ*pho13, *Δ*asc1, evolved		76	46	0.1	0.29	56.7	0.14	0.48	
XUSE	BY4741, xylA*3, TAL1, XKS1, Δgre3, *Δ*pho13, evolved		20	20	0.11	0.23	18.7	0.11	0.46	[12]
SR8N	D452-2*, Xyl1*, *Xyl2*, *Xyl3*, *Δ*ald6, noxE, evolved	Rich YP medium	70	40	0.19	0.52	47	0.22	0.431	[37]
SXA-R2P-E	BY4741, xylA*3, TAL1, XKS1, Δgre3, *Δ*pho13, evolved	Defined CSM medium	74.1	43.7	0.12	0.33	50	0.14	0.43	[11]
DXS	D452-2, mt*xyl1*, *xyl2*, *xyl3*	Rich YP medium	70	40	0.15	0.46	44.5	0.19	0.427	[38]
GLBRCY 128	NRRL YB-210 MATa, xylA, XYL3, TAL1, evolved	Rich YP medium	60	30	0.07	0.43	34	0.19	0.43	[39]
LF1	BSIF (diploid), *Δ*gre3, *Δ*pho13, xylA, XK, PPP, *MGT05196* ^N360F^, evolved	Rich YP medium	80	40	0.38	1.04	57	0.49	0.475	[31]
STXQ	Diploid strain of 36α2XpXpUN (Industrial ATCC 24860, ∆gre3, *xylA*, *XK*, PPP, evolved) with 39a2XoNK (ATCC 24860, ∆gre3, ∆cyc3, ∆ura3, *xylA*, *XK*, PPP, evolved)	Rich YP medium	162	95	0.42	1.13	120.6	0.53	0.48	[32]
CIBTS0735	Industrial CCTCC M94055 (diploid), *Δ*gre3, *xylA*, XKS1, TAL1, RPE1, TKL1, RKI1, GXF1, evolved	Rich YP medium	80	40	0.19	0.57	53	0.25	0.45	[40]
P5E49	Industrial NAPX37, *Δ*xyl1, *Δ*xyl2, *Δ*gre3*, xylA*, *Bgl1*, *Hxt7*, *Gxs1*, evolved	Rich YP medium	50	37	0.17	0.47	34.1	0.2	0.447	[41]
JX123_noxE	Industrial JHS200, xyl1, xyl2, xyl3, noxE.	Rich YP medium	70	40	0.18	0.5	47	0.21	0.43	[24]
424A (LNH-ST)	Industrial strain, *xyl1, xyl2, XKS1*	Rich YEP medium	70	40	–	–	45.6	–	0.43	[19]
36aS1.10.4	Industrial ATCC 24860, ∆gre3, *xylA*, *XK*, PPP, evolved	Defined YNB medium	62	38	0.2	0.46	41.07	0.21	0.42	[42]
MEC 1121	Industrial PE-2, *xyl1*, *xyl2*, *XKS1*, *TAL1*	Rich YP medium	38	27	–	–	19.6	–	0.31	[43]

^a^Fermentation conducted at 33 °C

### Co-fermentation performance was boosted up by elevating the fermentation temperature

**Fig. 4:**
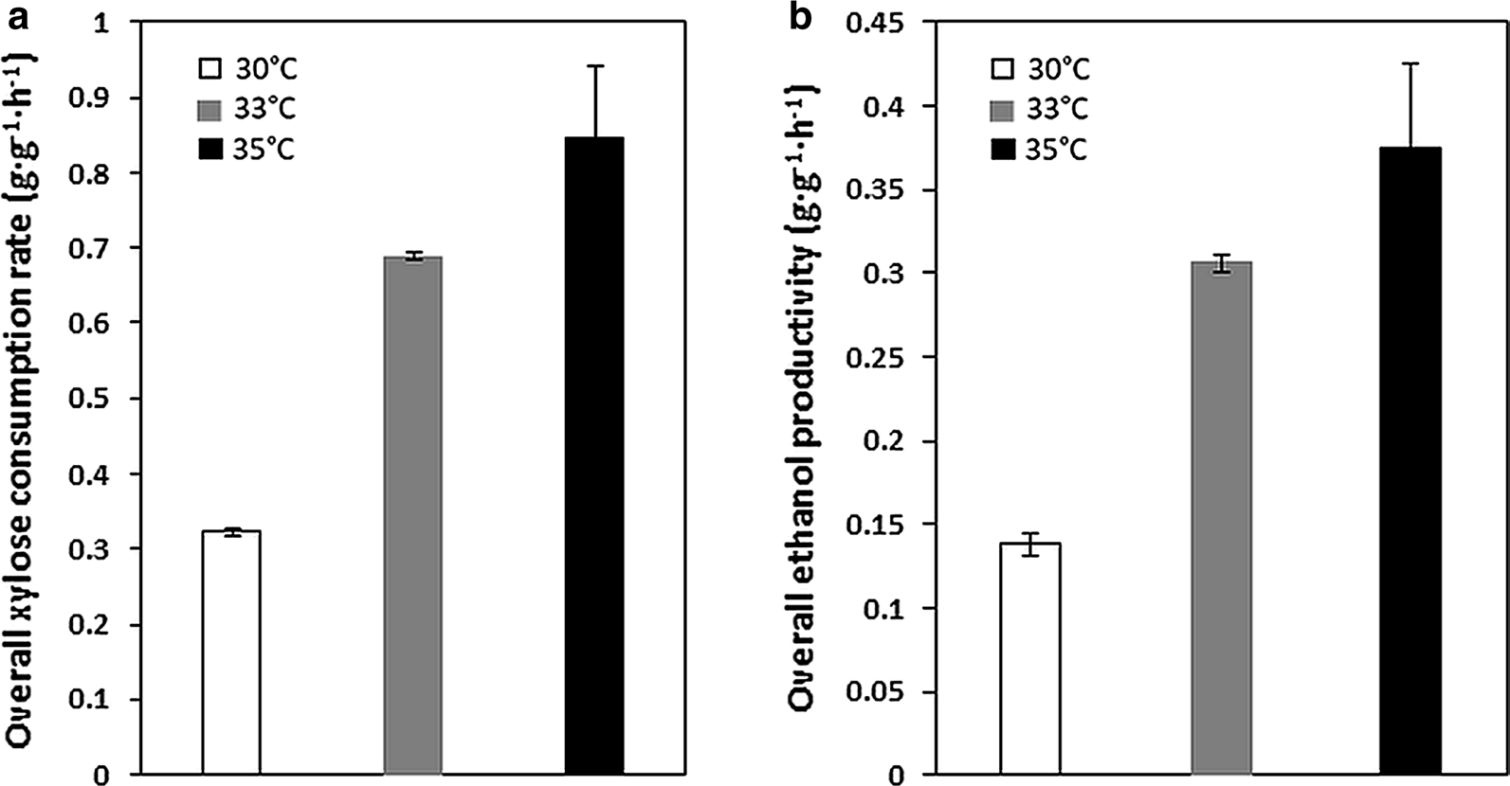
Xylose fermentation performance of XUSEA at 30 °C (while), 33 °C (gray), and 35 °C (black). **a** Xylose consumption rate and **b** ethanol productivity. Error bars represent the standard deviation of biological triplicates

Since xylose isomerization is an endothermic reaction, we conducted xylose fermentation at elevated temperatures ranging from 30 to 35 °C to accelerate the rate of the xylose isomerizing reaction and further improve the xylose fermentation efficiency. As reported previously, although the increased fermentation temperature would be more favorable for xylose isomerization, the cell viability issue could result in decreased fermentation performance [20]. Elevated fermentation temperature induces heat shock responses, such as cell cycle arrest, leading to reduced cell viability [21]. Accordingly, we set the fermentation temperature up to 35 °C. During low-cell-density xylose fermentation, the xylose consumption rate was improved by 2.2- and 2.7-fold at 33 °C and 35 °C (0.69 g g^−1^ h^−1^ and 0.85 g g^−1^ h^−1^, respectively) compared to that at 30 °C, respectively (0.32 g g^−1^ h^−1^) (Fig. 4a). With increased xylose consumption rate, ethanol productivity was also increased by 2.2- and 2.7-folds (0.14 g g^−1^ h^−1^, 0.31 g g^−1^ h^−1^, and 0.38 g g^−1^ h^−1^ at 30 °C, 33 °C, and 35 °C, respectively) demonstrating high-yield ethanol production phenotype was maintained even with increased xylose utilization rates (Fig. 4b). The improved fermentation performance resulted in significantly reduced fermentation time required for complete utilization of xylose from 72 to 45 h. During mixed-sugar fermentation of 20 g L^−1^ of glucose and 20 g L^−1^ of xylose, we persistently observed the superior xylose fermentation performance in terms of both xylose consumption and ethanol production rates at higher temperatures compared to those obtained at 30 °C (Additional file 3: Figure S3). Interestingly, increased sugar consumption was more clearly evident with xylose than with glucose, possibly due to the accelerated xylose isomerization reaction under increased temperature in complying with Le Chatelier’s principle. Lower cell growth during fermentation was observed at higher temperature (Additional file 4: Figure S4a, b), especially at 35 °C implying the weak thermotolerance of XUSEA. Moreover, as the fermentation performance at 35 °C was not significantly superior to that at 33 °C, further experiments were conducted at 33 °C, which requires less energy to maintain the temperature.

**Fig. 5:**
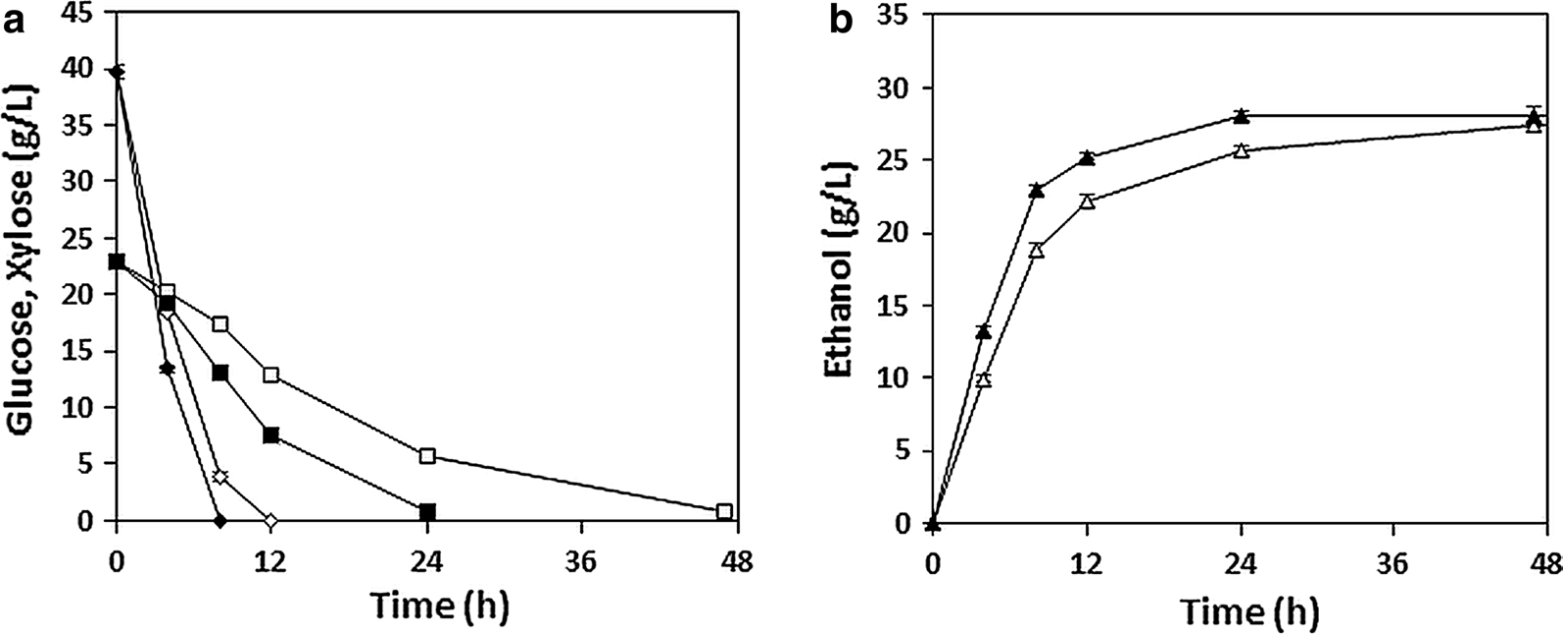
Micro-aerobic co-fermentation performance of XUSEA (40 g L^−1^ glucose and 20 g L^−1^ xylose) with a high cell density (initial OD_600_ of 20) at 30 °C (white) and 33 °C (black). **a** glucose (open rhombus) and xylose (open square) consumption, **b** ethanol (open triangle) production. Error bars represent the standard deviation of biological triplicates

To evaluate the co-fermentation performance of XUSEA during high-cell-density fermentation at an elevated temperature, we conducted mixed-sugar fermentation at 30 °C and 33 °C with an initial OD of 20. With increased cell density, XUSEA utilized 39.6 g L^−1^ of glucose and 22.8 g L^−1^ of xylose within 24 h (Fig. 5). Similar to the case of low-cell-density fermentation, a significant increase in sugar utilization was more apparent with xylose than with glucose. With an increased xylose consumption rate, the total fermentation time was reduced by half simply by increasing the fermentation temperature (48 h at 30 °C vs. 24 h at 33 °C). Notably, reduced cell growth was not observed with elevated temperature in the presence of glucose during co-fermentation (Additional file 4: Figure S4c), suggesting that the adverse effect of increased temperature on cell viability could be avoided during lignocellulosic bioethanol production using real biomass hydrolysates containing both glucose and xylose. Improved thermotolerance against elevated temperature was also reported previously in the presence of glucose, suggesting a protective effect of glucose supplementation under stress conditions [22].

### Efficient co-fermentation was achieved during lignocellulosic bioethanol production

**Fig. 6:**
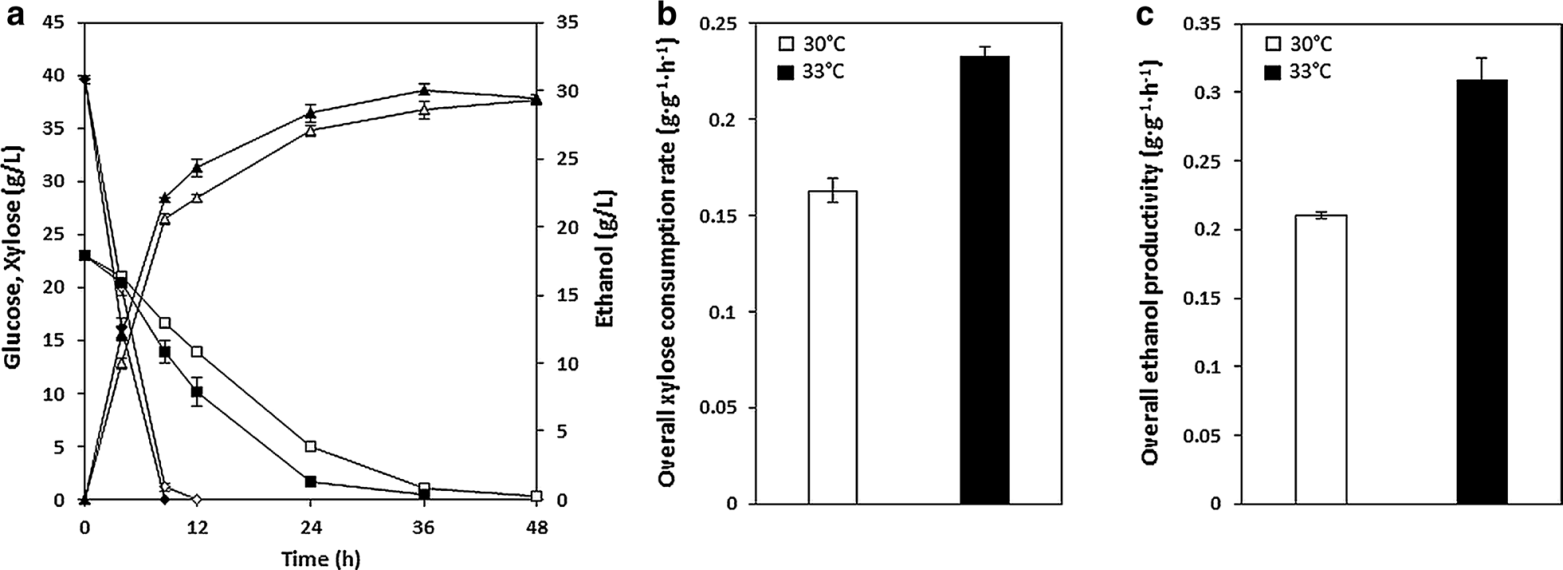
Co-fermentation performance of *Miscanthus* hydrolysate (40 g L^−1^ glucose and 20 g L^−1^ xylose) using XUSEA with a high cell density (initial OD_600_ of 20) at 30 °C (white) and 33 °C (black). **a** Sugar consumption and ethanol production: (open rhombus) glucose, (open square) xylose, (open triangle) ethanol. **b** Overall xylose consumption rate and **c** overall ethanol productivity at 30 °C (white) and 33 °C (black). Error bars represent the standard deviation of biological triplicates

Finally, we evaluated the co-fermentation performance of XUSEA using lignocellulosic hydrolysates of *Miscanthus* at both 30 °C and 33 °C. To clearly evaluate the co-fermentation performance of XUSEA, lignocellulosic bioethanol production was conducted without supplementation of enriched medium components such as yeast extracts, which are routinely added during lignocellulosic fermentation experiments to boost strain performance. As shown in Fig. 6a, XUSEA simultaneously utilized 39.6 g L^−1^ glucose and 23.1 g L^−1^ xylose, and produced 30.1 g L^−1^ of ethanol with a yield of 0.48 g g^−1^ (Table 2), within 24 h of fermentation at 33 °C. The elevated temperature resulted in a 44% increase in the xylose consumption rate (0.23 g g^−1^ h^−1^ vs. 0.16 g g^−1^ h^−1^) (Fig. 6b) and a 47% increase in the total sugar consumption rate (0.66 g g^−1^ h^−1^ vs. 0.45 g g^−1^ h^−1^). The ethanol production rate was also increased by 23% during lignocellulosic fermentation (Fig. 6c).

**Table 2:** Comparison of the hydrolysate fermentation performance among engineered xylose-utilizing *S. cerevisiae* strains

Strain	Description	Hydrolysate	Sugar (g L^−1^)		Overall xylose consumption rate (g g^−1^ h^−1^)	Overall total sugar consumption rate (g g^−1^ h^−1^)	Max. ethanol concentration (g L^−1^)	Overall ethanol productivity (g g^−1^ h^−1^)	Ethanol yield (g g^−1^)	References
			Glu.	Xyl.						
XUSEA^a^	BY4741, xylA*3, TAL1, XKS1, RPE1, *Δ*gre3, *Δ*pho13, *Δ*asc1, evolved	H_2_SO_4_-treated *Micanthus sacchariflorus Goedae*-*Uksae*	39.6	23.1	0.23	0.66	30.1	0.31	0.48	This study
XUSEA	BY4741, xylA*3, TAL1, XKS1, RPE1, *Δ*gre3, *Δ*pho13, *Δ*asc1, evolved	H_2_SO_4_-treated *Micanthus sacchariflorus Goedae*-*Uksae*	39.6	23.1	0.16	0.45	29.3	0.21	0.47	
XUSAE57	BY4741, xylA*3, TAL1, XKS1, RPE1, *Δ*gre3, *Δ*pho13, evolved, acetic acid tolerance	Diluted acid-treated sugarcane bagasse	26.2	27.6	0.1	0.21	25	0.1	0.49	[25]
SXA-R2P-E	BY4741, xylA*3, TAL1, XKS1, Δgre3, *Δ*pho13, evolved	Diluted acid- treated rice straw	27.7	20.4	0.05	0.13	20.7	0.06	0.46	[11]
		Diluted acid- treated oak	26.8	16	0.04	0.11	17.7	0.05	0.43	
DXS	D452-2, mt*xyl1*, *xyl2*, *xyl3*	Silver grass hydrolysate	92	32	–	–	50.7	–	0.43	[38]
LF1	BSIF (diploid), *Δ*gre3, *Δ*pho13, xylA, XK, PPP, *MGT05196* ^N360F^, evolved	SECS hydrolysate	86.6	39.1	0.18	0.61	49	0.24	0.413	[31]
		SPPR hydrolysate	55	23.8	0.29	0.97	31	0.38	0.416	
GLBRCY 128	NRRL YB-210 MATa, xylA, XYL3, TAL1, evolved	AFEX-treated corn stover	60	30	0.11	0.57	31	0.23	0.39	[39]
36aS1.10.4	Industrial ATCC 24860, ∆gre3, *xylA*, *XK*, PPP, evolved	Wheat straw	82.66	43.96	0.19	0.55	54.11	0.24	0.44	[42]
		Oil palm empty fruit hydrolysate	83.17	43.56	0.15	0.44	50.36	0.17	0.41	
JX123_noxE	Industrial JHS200, xyl1, xyl2, xyl3, noxE.	Silver grass hydrolysate	106	32	0.23	1.02	55.5	0.41	0.433	[24]
STXQ	Diploid strain of 36α2XpXpUN (Industrial ATCC 24860, ∆gre3, *xylA*, *XK*, PPP, evolved) with 39a2XoNK (ATCC 24860, ∆gre3, ∆cyc3, ∆ura3, *xylA*, *XK*, PPP, evolved)	Oil palm empty fruit hydrolysate	41.81	30	–	–	28.4	–	0.42	[32]

^a^Fermentation conducted at 33 °C

## Discussion

Development of a production host for lignocellulosic bioconversion requires the efficient co-fermentation of glucose and xylose, two primary sugars present in a lignocellulosic hydrolysate. However, when *S. cerevisiae* utilizes both sugars, sequential xylose fermentation with low conversion rate occurs leading to reduced volumetric ethanol productivity with prolonged fermentation time [23]. In this study, we demonstrated highly efficient co-fermentation of lignocellulosic hydrolysates by a newly engineered *S. cerevisiae*, XUSEA, with improved xylose utilization capacity. By reinforcing xylose catabolism and elevating fermentation temperature, significantly improved glucose/xylose co-fermentation was achieved resulting in among the highest ethanol yield and productivity during lignocellulosic bioethanol production. With the improved xylose catabolic pathway, XUSEA showed over twofold higher xylose consumption and ethanol production rates than those of its parent strain. The highly efficient co-fermentation performance of XUSEA was maintained even with industrial-scale high-level sugar medium. XUSEA completely converted 76 g L^−1^ of glucose and 46 g L^−1^ of xylose into ethanol with an yield of 0.5 g g^−1^ (98% of theoretical maximum of 0.51 g g^−1^) within 72 h. The slight increase in fermentation temperature also considerably boosted the co-fermentation performance of XUSEA resulting in over twofold increased xylose consumption and ethanol production rates. The highly efficient co-fermentation performance was confirmed during lignocellulosic bioethanol production resulting in the ethanol yield of 0.48 g g^−1^ and productivity of 0.31 g g^−1^ h^−1^, among the highest values reported to date (Table 2). Lee et al. reported ethanol productivity of 0.41 g g^−1^ h^−1^ with a yield of 0.433 g/g by oxidoreductase-based xylose-utilizing strain during silver grass hydrolysate fermentation with a 3.3:1 glucose/xylose ratio [24]. Given that the xylose consumption rate of XUSEA was the same as that of the industrial strain used by Lee et al. [24], the higher ethanol productivity could be due to the significant portion of glucose, which can support much higher ethanol productivity than xylose, in the hydrolysates and the industrial background of the strain. It should also be noted that the xylose-utilizing strains with the oxidoreductase-based pathway have advantages in xylose utilization rate, whereas those with the isomerase-based pathway have benefits in product yield. The same xylose utilization rate of isomerase-based XUSEA with the oxidoreductase-based industrial strain reported by Lee et al. clearly shows superior performance of XUSEA in terms of not only ethanol yield but also productivity. The ethanol yield of XUSEA, 0.48 g g^−1^, during lignocellulosic hydrolysates was the second highest following the one reported in the sister strain of XUSEA, XUSAE57, in which acetate tolerance was improved through evolutionary engineering [25]. However, almost threefold higher ethanol productivity of XUSEA over XUSAE57 clearly shows XUSEA could serve as a more promising production host for economically viable lignocellulosic biorefinery in an industrial process.

To improve xylose conversion efficiency, XUSEA was engineered based on the one of the best xylose fermenting strains of XUSE which demonstrated the highest yield during lignocellulosic bioethanol production [25] with its cofactor-neutral isomerase-based pathway. Though XUSE simultaneously converted both glucose and xylose to the target product, the low xylose conversion rate was limited to the overall co-fermentation efficiencies suggesting the remaining challenges of further enhancement in xylose fermentation performance to truly achieve economically feasible lignocellulosic biorefinery. With additional copy of xylose isomerase (xylA*3) and overexpressing *RPE1* gene in PP pathway, XUSEA showed significantly improved co-fermentation efficiency especially in terms of xylose conversion rates. Previously, multiple copies of xylose isomerase gene integration and/or intensive overexpression of PP pathway genes have been reported to improve xylose fermentation performance [16, 17, 26–28]. Specifically, overexpression of genes involved in the pentose phosphate (PP) pathway such as transketolase (*TKL1*), ribulose-5-phosphate 3-epimerase (*RPE1*), and ribose 5-phosphate ketol-isomerase (*RKI1*) has been commonly implemented to develop xylose-utilizing strains [16, 17]. Interestingly, XUSE presented remarkable xylose fermentation performance even without intensive engineering of the PP pathway [12], which could be due to its different cellular network rearranged by the introduced cofactor-neutral isomerase-based xylose catabolic pathway [12]. This implied the possibility for further improvement in the xylose fermentation performance of XUSE through minimal PP pathway engineering. By harnessing the power of DNA assembly and growth-based strategies, we were able to effectively identify the critical overexpression target in PP pathway, *RPE1*, and improved xylose fermentation performance even with minimal engineering. This approach not only save engineering efforts for strain construction dealing with multiple overexpression targets but also more effectively guarantee the intended effects without the association of unnecessary genes. In addition, by integrating overexpression cassette into the right spot of *ASC1* locus, which turn to be non-functional, through marker-free genome editing system of CRISPR-Cas9, XUSEA still have plentiful room for further engineering. This makes XUSEA attractive host for lignocellulosic biorefinery not limiting its product only to bioethanol.

This study not only provides an efficient co-fermenting strain of XUSEA enabling high-yield bioethanol production from lignocellulosic hydrolysates, but also offers an effective fermenter-operating strategy to further improve the xylose fermentation performance. XUSEA harbors heterologous xylose isomerase mediating one-step isomerization reaction in which xylose is converted to xylulose. As endothermic reaction, we hypothesized that the reaction rate could be accelerated by elevating fermentation temperature based on Le Chaterlier’s principle. In vitro effect of varying temperature on xylose isomerization was investigated by Roman et al. [15]. Besides, Cunha et al. reported the consequences of conducting fermentation at high temperature (30 °C vs. 40 °C) in *S. cerevisiae* [29]. To the best of our knowledge, however, the beneficial effects of elevated temperature on xylose fermentation and mixed-sugar fermentation in xylose isomerase-based *S. cerevisiae* have never been evaluated. To this end, we evaluated the improvement of xylose fermentation efficiency by cultural temperatures ranging from 30 to 35 °C. Although the higher temperature could increase xylose isomerization rate in XUSEA, the cell viability issue set the limit of the beneficial temperature to 33 °C for co-fermentation of glucose and xylose. When cells are cultured at elevated temperatures, heat shock responses are induced, leading to cellular events such as cell cycle arrest in the G1 phase, which may be associated with lower cell growth [21]. Since xylose-utilizing strains tend to be more sensitive toward stress conditions [1], the increased temperature could have a more severe effect on the cellular fitness of XUSEA. During the process of fermentation, we observed reduced cell viability at higher temperatures, especially at 35 °C. Even though elevated fermentation temperatures adversely affect cell viability, the positive effect of elevated temperatures has also been reported previously. Jones et al. reported a marked decrease in respiration and an increase in pyruvate levels in yeast cells at high temperatures [30]. The increased abundance of cytosolic pyruvate could favor ethanol fermentation, while low respiratory capacity could limit biomass synthesis. This suggests the possible reason for accelerated ethanol production despite reduced cell growth. During co-fermentation at elevated temperature, XUSEA showed significantly improved performance resulting in the highest ethanol yield with sufficiently high productivity among previously reported strains (Table 1). Higher ethanol productivities have been reported with two industrial strains, STXQ and LF1. This could be attributed to the robustness of the industrial strain background and supplementation with nutrient-rich YP medium, which might better support yeast cell growth and fermentation performance [31, 32]. A slight increase in fermentation temperature successfully boosted the improved xylose fermentation performance provided by genetic engineering, thus resolving a critical limiting factor for realizing efficient lignocellulosic bioethanol production.

## Conclusions

In this study, we achieved efficient bioethanol production from lignocellulosic hydrolysates with high yield and productivity by reinforcing xylose catabolism and increasing the fermentation temperature. Industrially relevant lignocellulosic fermentation at high temperature can offer additional practical benefits, including cooling cost reduction, prevention of contamination, and simultaneous hydrolysis and saccharification [33]. Moreover, since XUSEA was minimally engineered using the marker-free CRISPR-Cas system, this strain can easily be further engineered to improve its co-fermentation performance or thermotolerance, and to expand the product profile. Therefore, XUSEA could serve as a platform strain for efficient production of fuels and chemicals from lignocellulosic biomass and thus promote the expansion of lignocellulosic biorefinery.

## Methods

### Strains, plasmids, and culture conditions

The yeast strains used in this study were isogenic to *S. cerevisiae* S288C BY4741 and are listed in Additional file 5: Table S1. The yeast strains were routinely cultivated at 30 °C in yeast synthetic complete (YSC) medium composed of xylose (or glucose), 6.7 g L^−1^ of a yeast nitrogen base (Difco, Detroit, MI, USA), and 0.79 g L^−1^ complete synthetic medium (CSM; MP Biomedicals, Solon, Ohio, USA). *Escherichia coli* DH10β was used for DNA manipulation and expression of recombinant genes, which was cultured at 37 °C in Luria–Bertani medium supplemented with 100 μg mL^−1^ ampicillin. All yeast and bacterial cultivations were performed in orbital shakers at 200 rpm.

### Selection of an engineering target in the PP pathway

A library of different combinations of various promoters (P) and terminators (T) harboring three PP pathway genes, *TKL1*, *RPE1,* and *RKI1* (e.g., P1-TKL1-T1-P2-RPE1-T2-P3-RPE1-T3), was constructed in a p416 backbone vector using the DNA assembler method as reported previously [18]. DNA fragments including three different promoters (GPDp, TEFp, and CYC1p) and terminators (CYC1t, SPG5t, and PRM9t) and the three PP genes (Additional file 6: Figure S5) were amplified by polymerase chain reaction (PCR) from genomic DNA of *S. cerevisiae* BY4741 with primers including flanking regions homologous to adjacent fragments. After purification, all DNA fragments (300 ng each) were co-transformed with the PvuII-digested p416 backbone vector (500 ng) into the XUS *S. cerevisiae* strain harboring a xylose isomerase pathway through electroporation (Gene Pulser Xcell™ Electroporation System, Bio-Rad). The obtained transformants were then cultured in 20 mL of CSM-Ura liquid medium supplemented with 20 g L^−1^ of xylose and serially transferred into fresh liquid medium with a 0.05% inoculum size four times to confer high growth ability for the dominant population on xylose via improved xylose catabolism. The cells were spread on CSM-Ura plates and the 100 largest colonies were picked out and subjected to three rounds of growth-based selection using TECAN-based, culture tube-based, and serum bottle-based methods as previously described [12]. Finally, the most optimal combination was identified by sequencing the plasmid extracted from the best-performing strain selected based on the xylose fermentation performance.

### Development of a glucose–xylose co-fermenting strain, XUSEA

A CRISPR-Cas9 genome editing approach was used for obtaining strain XUSE, using the plasmids listed in Additional file 5: Table S1. Specifically, p413-Cas9 was modified from the p414-TEF1p-*Cas9*-CYC1t plasmid (Addgene plasmid #43802) by replacing the selection marker, and p426gASC1 expressing gRNA targeting *ASC*1 was constructed based on a gRNA expression plasmid (Addgene #43803) [34] by replacing a 20-nt target sequence with a sequence targeting *ASC1* (CCAAGATGAAGTTTTCTCTT). The donor DNA fragment containing an overexpression cassette of xylA*3 [35] and *RPE1* (GPDp-xylA*3-PRM9t-GPDp-*RPE1*-SPG5t) flanking 100 bp of the homology arms targeting sequences upstream and downstream of *ASC1* was cloned into the pUC19 plasmid, resulting in p-dASC1, which was digested with BamHI to prepare the donor DNA cassette. The p426-gASC1 and donor DNA were then co-transformed into XUSE harboring a Cas9-expressing plasmid by electroporation, resulting in strain XUSEA. Successful integration of the desired cassette was verified by PCR-based diagnosis from genomic DNA extracted from transformant colonies cultured in CSM-Ura-His liquid medium. The ultimate strain, XUSEA, was then subjected to subculture on CSM supplemented with 20 g L^−1^ glucose for plasmid rescue.

### Fermentation

For seed culture, cells were inoculated in YSC medium containing 2% glucose. The cells were then transferred to fresh YSC medium containing 2% xylose with an inoculum size of 5% and grown aerobically in flasks for 1.5–2 days. Precultured cells were harvested and inoculated into fresh YSC medium for fermentation. The pH of the fermentation medium was maintained at 5.0 by adding 100 mM phthalate buffer. Microaerobic fermentation was carried out in 125-mL serum bottles with a final working volume of 40 mL at a low cell density with initial optical density (OD) of 0.2 or high cell density with initial OD of 20. The serum bottles were capped with rubber stoppers with a needle for carbon dioxide release during fermentation. To determine the effect of increased temperature, the main culture fermentation was conducted at 30 °C, 33 °C, and 35 °C, respectively.

Lignocellulosic hydrolysates, *Miscanthus sacchariflorus Goedae*-*Uksae 1*, treated with diluted acids and detoxified with activated carbon, defined as the Saccharomate hydrolysate, were purchased from SugarEn (Gyeonggi-do, Korea). The culture medium for hydrolysate fermentation contained 39.6 g L^−1^ glucose, 23.1 g L^−1^ xylose, 6.7 g L^−1^ of a yeast nitrogen base (Difco, Detroit, MI, USA), 0.79 g L^−1^ CSM, and 100 mM phthalate buffer to maintain the pH at 5.0.

### Analytical methods

Cell growth was analyzed by measuring the OD at 600 nm with a spectrometer (Cary 60 Bio UV–Vis, Agilent Technologies, USA), in which an OD at 600 nm of 1 was calculated to correspond to 0.17 g cells L^−1^ based on Jin et al. [36]. Concentrations of glucose and xylose were analyzed by a high-performance liquid chromatography system (HPLC 1260 Infinity, Agilent Technologies, Palo Alto, CA, USA) equipped with a refractive index detector using a Hi-Plex H column (Agilent Technologies). The system was operated with 5 mM H_2_SO_4_ as the mobile phase at a flow rate of 0.6 mL min^−1^ and a column temperature of 65 °C. The ethanol concentration was detected using a gas chromatography (Agilent Technologies) equipped with a flame ionization detector using an HP-INNOWax polyethylene glycol column (30 m × 0.25 µm × 0.25 µm).

## Supplementary information

**Additional file 1: Figure S1.** Growth of PP pathway-harboring candidate strains (XUSA) on xylose during TECAN-based selection; the best-performing strain was found to be expressing *RPE1*. Relative OD_600_ value of the XUSA strains was calculated based on the OD_600_ value of the XUS strain at the stationary phase. Error bars represent the standard deviation of biological triplicates.

**Additional file 2: Figure S2.** Microaerobic fermentation of xylose (20 g L^−1^) with the XUSEA (black circles, solid line) and XUSE (white circles, dashed line) strains. a. xylose utilization, b. ethanol production. Error bars represent the standard deviation of biological triplicates.

**Additional file 3: Figure S3.** Fermentation performance of glucose (20 g L^−1^) and xylose (20 g L^−1^) using XUSEA strain at different culture temperatures: 30 °C (white), 33 °C (gray), 35 °C (black). a. Xylose consumption rate, b. Ethanol productivity. Error bars represent standard deviation of biological triplicates.

**Additional file 4: Figure S4.** Cell growth of the XUSEA strain during fermentation at different culture temperatures: 30 °C (white), 33 °C (gray), 35 °C (black). (a) low-cell-density fermentation with 20 g L^−1^ xylose; (b) low-cell-density fermentation with 20 g L^−1^ glucose and 20 g L^−1^ xylose; (c) high-cell-density fermentation with 40 g L^−1^ glucose and 20 g L^−1^ xylose. Error bars represent standard deviation of biological triplicates.

**Additional file 5: Table S1.** Strains and plasmids used in this study.

**Additional file 6: Figure S5**: Schematic illustration of the library construction of different combinations of various promoters and terminators harboring three PP pathway genes, *TKL1*, *RPE1,* and *RKI1*, using the DNA assembler method as reported previously.

## Data Availability

The datasets used and/or analyzed during the current study are available from the corresponding author upon reasonable request.
