# Supplementary figures and images for: Improved simultaneous co-fermentation of glucose and xylose by Saccharomyces cerevisiae for efficient lignocellulosic biorefinery
Source: Biotechnol Biofuels. 2020 Jan 22;13:12. doi: 10.1186/s13068-019-1641-2 (PMC6975041; doi:10.1186/s13068-019-1641-2)

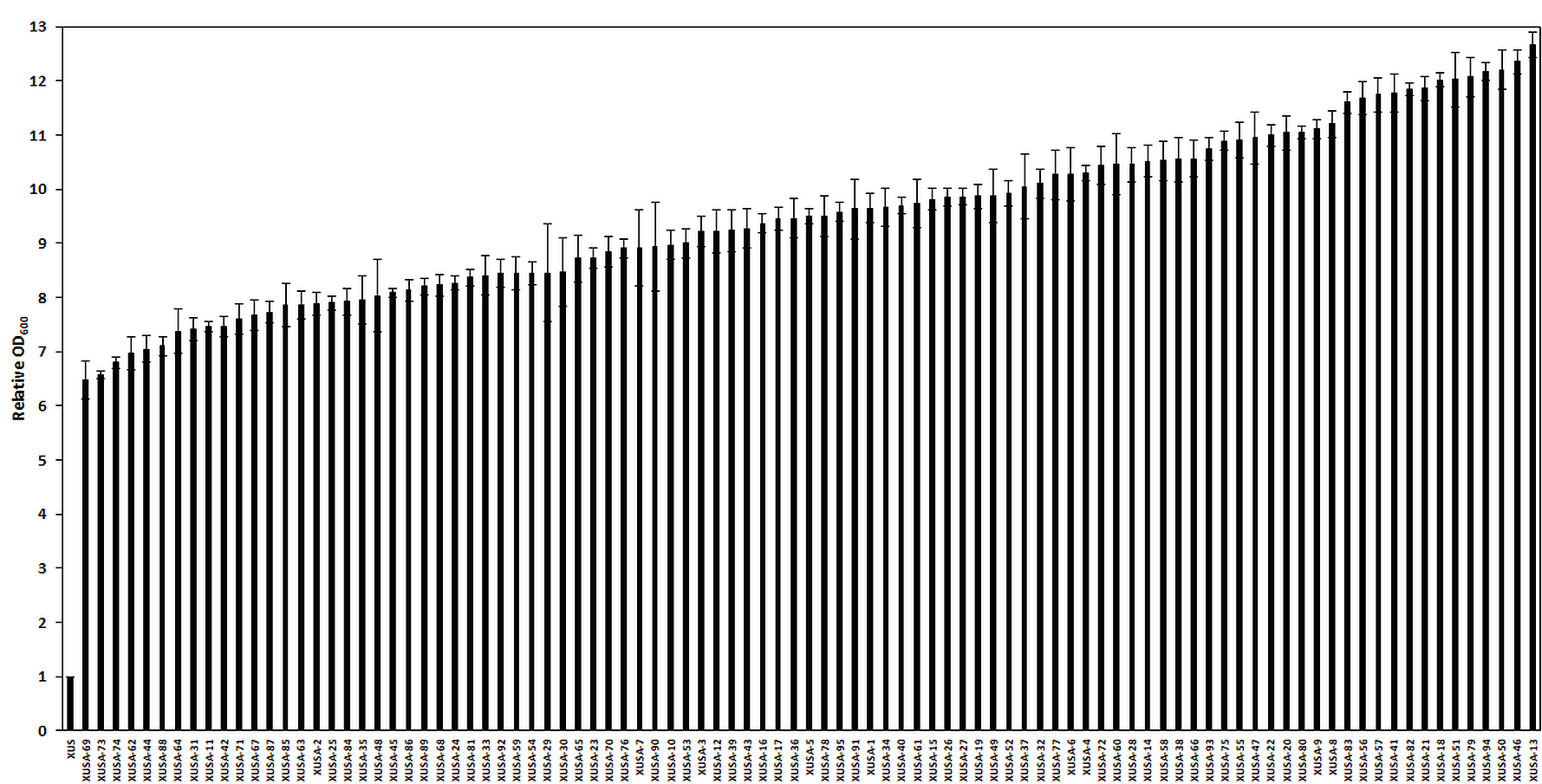

Supplement: Supplementary file 1 — Additional file 1: Figure S1. Growth of PP pathway-harboring candidate strains (XUSA) on xylose during TECAN-based selection; the best-performing strain was found to be expressing RPE1. Relative OD600 value of the XUSA strains was calculated based on the OD600 value of the XUS strain at the stationary phase. Error bars represent the standard deviation of biological triplicates. [file 13068_2019_1641_MOESM1_ESM.tif]

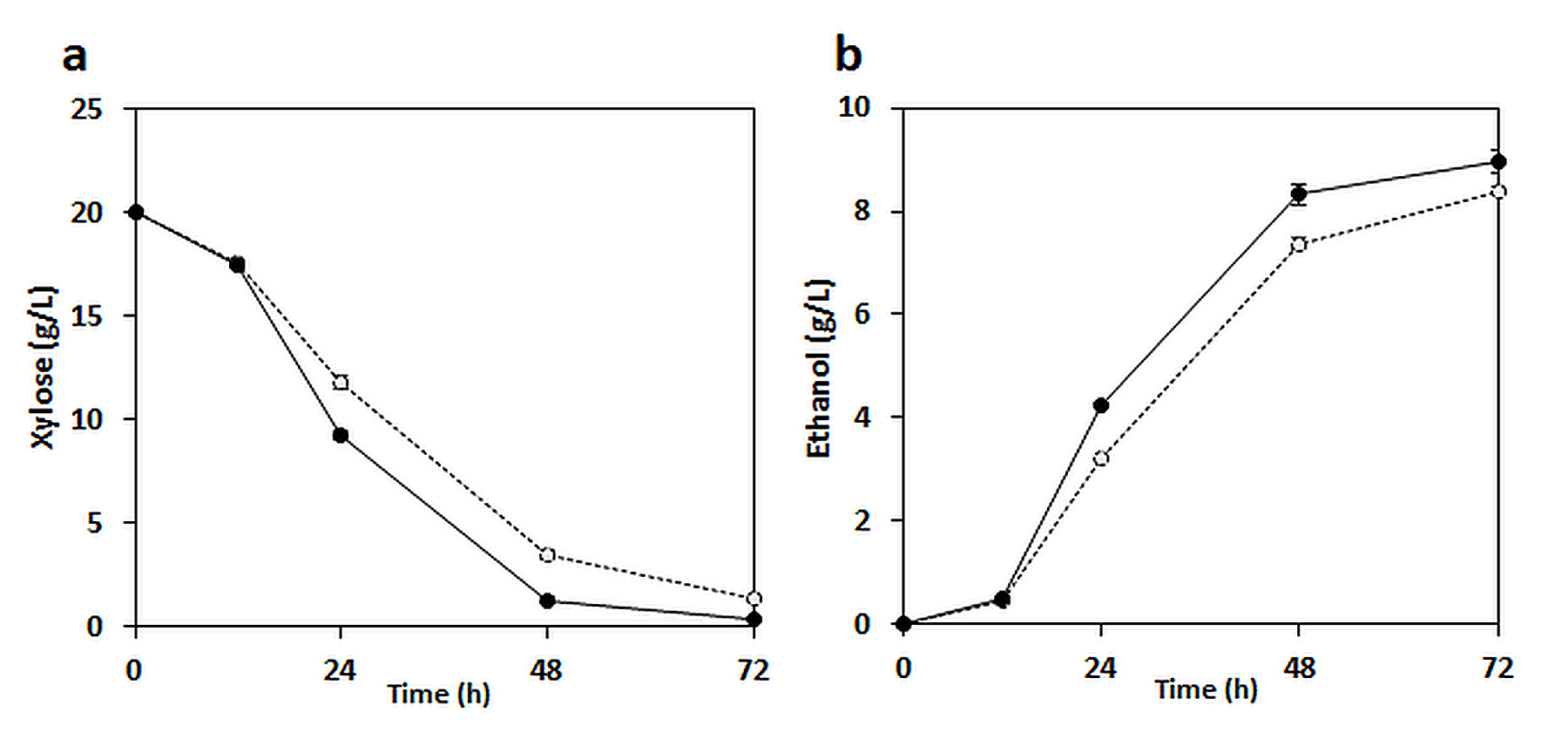

Supplement: Supplementary file 2 — Additional file 2: Figure S2. Microaerobic fermentation of xylose (20 g L−1) with the XUSEA (black circles, solid line) and XUSE (white circles, dashed line) strains. a. xylose utilization, b. ethanol production. Error bars represent the standard deviation of biological triplicates. [file 13068_2019_1641_MOESM2_ESM.tif]

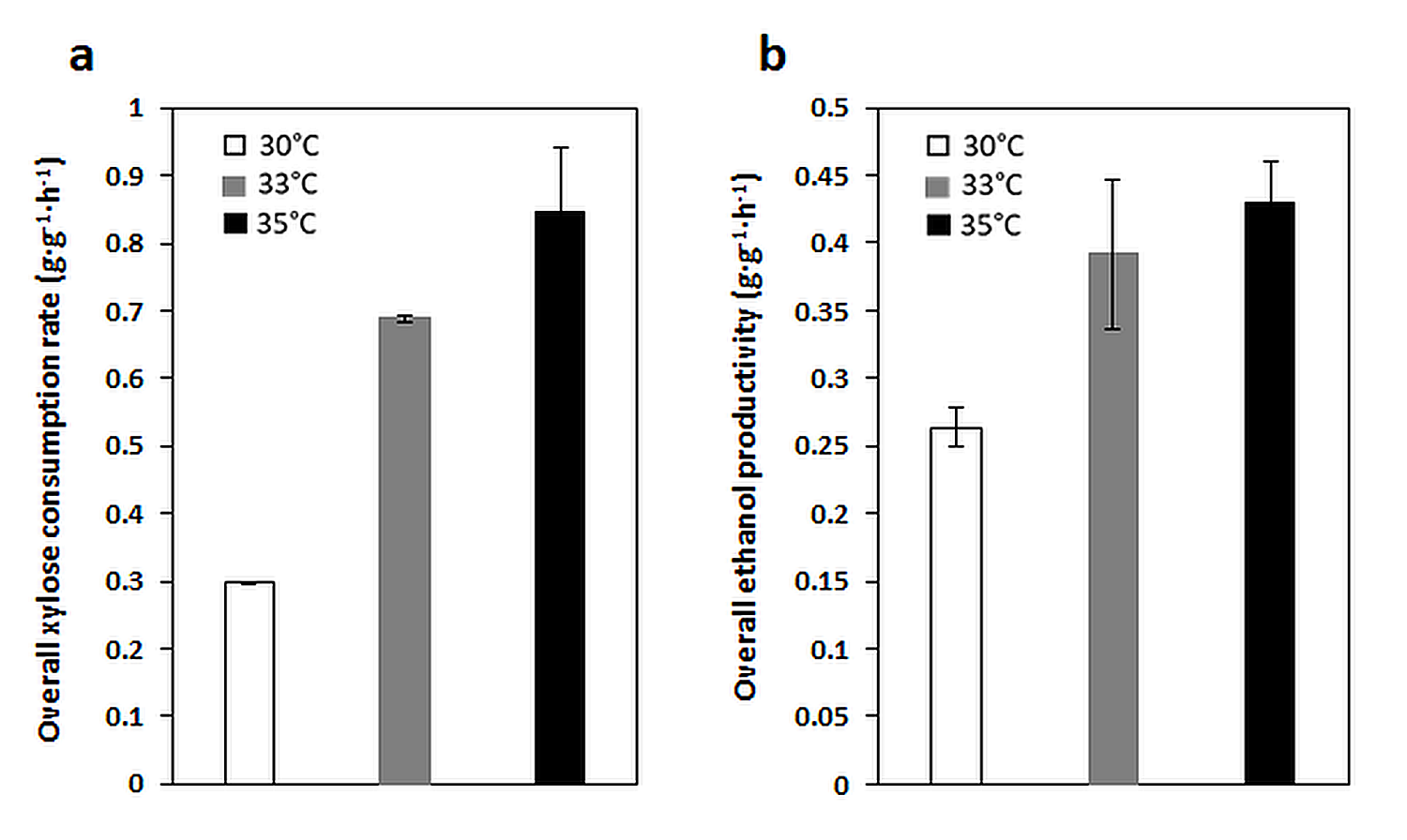

Supplement: Supplementary file 3 — Additional file 3: Figure S3. Fermentation performance of glucose (20 g L−1) and xylose (20 g L−1) using XUSEA strain at different culture temperatures: 30 °C (white), 33 °C (gray), 35 °C (black). a. Xylose consumption rate, b. Ethanol productivity. Error bars represent standard deviation of biological triplicates. [file 13068_2019_1641_MOESM3_ESM.tif]

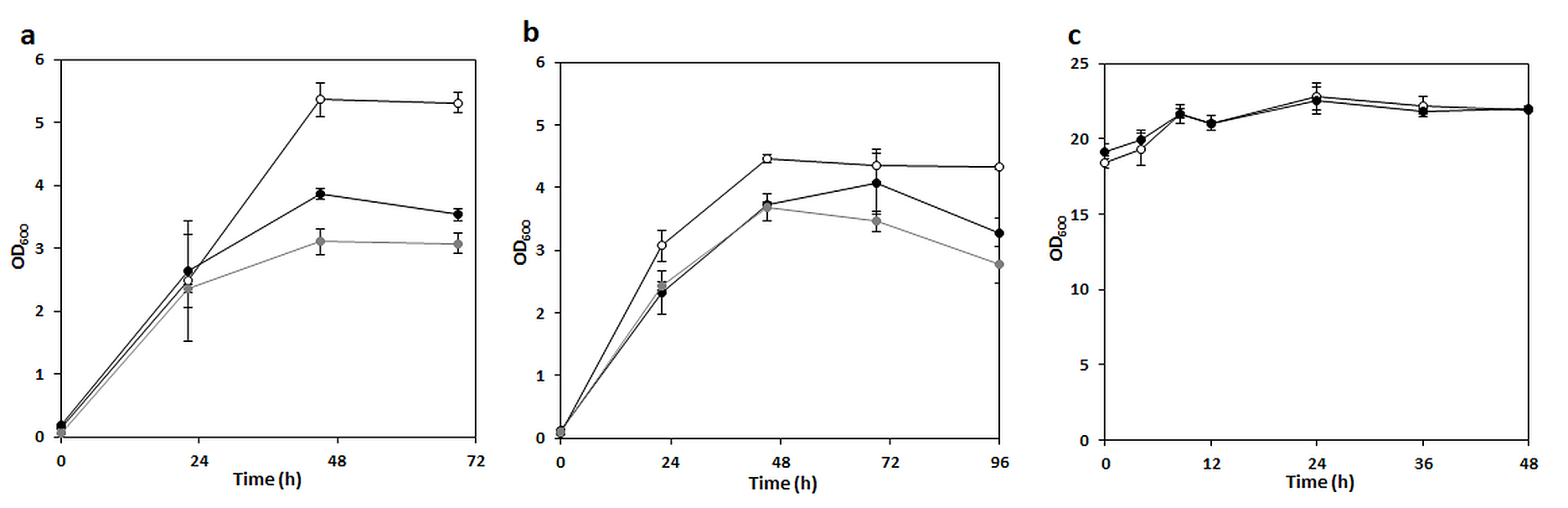

Supplement: Supplementary file 4 — Additional file 4: Figure S4. Cell growth of the XUSEA strain during fermentation at different culture temperatures: 30 °C (white), 33 °C (gray), 35 °C (black). (a) low-cell-density fermentation with 20 g L−1 xylose; (b) low-cell-density fermentation with 20 g L−1 glucose and 20 g L−1 xylose; (c) high-cell-density fermentation with 40 g L−1 glucose and 20 g L−1 xylose. Error bars represent standard deviation of biological triplicates. [file 13068_2019_1641_MOESM4_ESM.tif]

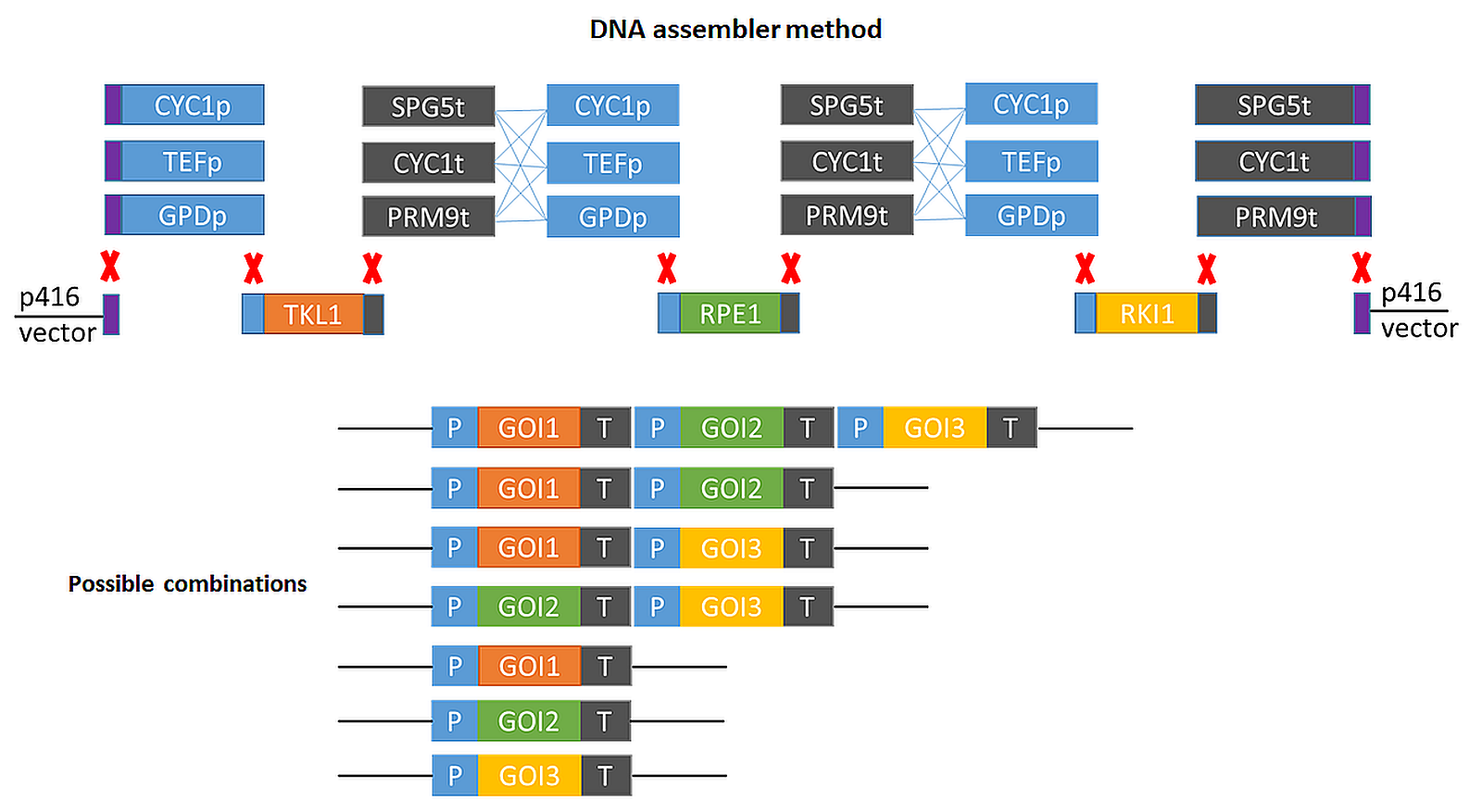

Supplement: Supplementary file 6 — Additional file 6: Figure S5: Schematic illustration of the library construction of different combinations of various promoters and terminators harboring three PP pathway genes, TKL1, RPE1, and RKI1, using the DNA assembler method as reported previously. [file 13068_2019_1641_MOESM6_ESM.tif]
