# Supplementary material for: Improved simultaneous co-fermentation of glucose and xylose by Saccharomyces cerevisiae for efficient lignocellulosic biorefinery
Source: Biotechnol Biofuels. 2020 Jan 22;13:12. doi: 10.1186/s13068-019-1641-2 (PMC6975041; doi:10.1186/s13068-019-1641-2)
Supplement: Supplementary file 5 — Additional file 5: Table S1. Strains and plasmids used in this study. [file 13068_2019_1641_MOESM5_ESM.docx]

**Table S1: Strains and plasmids used in this study**

| **Strains** | **Description** | **Reference** |
| --- | --- | --- |
| XUS | BY4741, *gre3*:: GPDp-*xylA*3*-PRM9t-TEFp-*XKS1*-CYC1t, *pho13*:: GPDp-*xylA*3*-PRM9t-TEFp-*TAL1*-CYC1t | [12] |
| XUSE | BY4741, *gre3*:: GPDp-*xylA*3*-PRM9t-TEFp-*XKS1*-CYC1t, *pho13*:: GPDp-*xylA*3*-PRM9t-TEFp-*TAL1*-CYC1t, evolved |  |
| XUSEA | XUSE, *asc1*:: GPDp-*xylA*3*-PRM9t-GPDp-*RPE1*-SPG5t | This study |
| **Plasmids** | **Description** |  |
| pRPE1 | p416 GPDp-RPE1-SPG5t | This study |
| p-dASC1 | pUC19 lHA- GPDp-*xylA*3*-PRM9t-GPDp-*RPE1*-SPG5t-rHA  Left and right homology arm of 100bp with *ASC1* locus |  |
| p413Cas9 | p413 TEFp- Cas9- CYC1t |  |
| p426gASC1 | p426 SNR52p-gRNA.ASC1-SUP4t targeting *ASC1* gene |  |
